# Rapid quantification and validation of etomidate and dexmedetomidine blood concentrations in rats using a novel portable mass spectrometer

**DOI:** 10.3389/fvets.2025.1696093

**Published:** 2025-10-29

**Authors:** Sicheng Liu, Xiaoxiao Li, Deying Gong, Yanhua Huang, Wensheng Zhang

**Affiliations:** ^1^Department of Anesthesiology, West China Hospital, Sichuan University, Chengdu, China,; ^2^Laboratory of Anesthesia and Critical Care Medicine, National-Local Joint Engineering Research Centre of Translational Medicine of Anesthesiology, West China Hospital, Sichuan University, Chengdu, China

**Keywords:** rapid analysis, mass spectrometer, HPLC-MS, etomidate, dexmedetomidine, rat

## Abstract

In clinical anesthesia, precise dosing depends on feedback from blood drug concentrations. However, rapid tools for measuring these concentrations are currently limited. This study evaluated the capability of a novel portable mass spectrometer (CELL) to rapidly quantify etomidate and dexmedetomidine in rat plasma. The 50% effective dose (ED_50_) of both drugs was determined using the up-and-down method. CELL's methodology was validated according to standard guidelines. Linear regression and intraclass correlation coefficient (ICC) analyses were conducted to assess the consistency between CELL and HPLC-MS measurements. The ED_50_ of etomidate was found to be 0.9 mg/kg and that of dexmedetomidine was 21.1 μg/kg in rats. For etomidate, CELL demonstrated linearity in the range of 210–2,000 ng/ml (*y* = 427.0*x* – 30,526, *R*^2^ = 0.995) with a limit of quantitation (LOQ) of 210 ng/ml. For dexmedetomidine, linearity was observed in the range of 6–1,000 ng/ml (*y* = 14,669*x* + 109,875, *R*^2^ = 0.997) with an LOQ of 6 ng/ml. In the mixed group, etomidate showed linearity in the range of 100–2,000 ng/ml (*y* = 431.4*x* + 11,864, *R*^2^ = 0.996) with an LOQ of 100 ng/ml, while dexmedetomidine exhibited linearity in the range of 5–1,000 ng/ml (*y* = 14,141*x* + 52,430, *R*^2^ = 0.997) with an LOQ of 5 ng/ml. A strong linear correlation was observed between CELL and HPLC-MS. These results indicate that CELL provides excellent performance in the rapid and simultaneous quantification of both drugs, supporting real-time monitoring to meet diverse clinical requirements. This approach has strong potential for point-of-care (POC) monitoring in perioperative veterinary and human anesthesia.

## 1 Introduction

To address the challenge of controlling the depth of anesthesia, therapeutic drug monitoring (TDM) applies pharmacokinetic principles by measuring drug concentrations in the blood, enabling individualized and precise dosing to maintain drug levels within a target therapeutic range—thereby optimizing efficacy while minimizing toxicity (1, 2). For example, target-controlled infusion (TCI) systems adjust infusion rates based on real-time plasma drug concentration feedback to maintain a preset target concentration (3). Furthermore, in outpatient or ward settings, the rapid determination of plasma drug concentrations is essential for assessing patient physiological or pathological status and facilitating timely adjustments to medication strategies. Among the main anesthetics used, etomidate, known for its rapid onset and minimal cardiovascular effects, is commonly used for anesthetic induction (4). Dexmedetomidine, due to its unique profile that provides sedation, analgesia, anxiolysis, and minimal respiratory depression, is widely used for sedation in intensive care units, as an adjunct for intraoperative sedation, and as a supplement to regional anesthesia (5, 6). However, achieving precise control over depth of anesthesia with these agents during clinical use remains challenging. Etomidate may cause dose-dependent suppression of adrenocortical function and myoclonus (7–9). Dexmedetomidine can induce significant hemodynamic fluctuations and may lead to delayed recovery at higher doses (10, 11). These adverse effects not only compromise patient safety and comfort but also increase the complexity of perioperative management. Although currently, high-performance liquid chromatography-mass spectrometry (HPLC-MS) serves as the gold standard for the quantitative analysis of anesthetic drugs in complex biological matrices such as plasma (12). Although HPLC-MS offers high sensitivity, high specificity, and a broad dynamic range, its application is subject to some limitations such as complex usage, which makes it difficult to meet the clinical demands of point-of-care (POC) testing (13, 14).

In animal experimental research, real-time monitoring of the depth of anesthesia and maintaining appropriate plasma drug concentrations are of vital importance. The aim is to ensure effective anesthesia while minimizing the risk of adverse drug reactions. Animal models represented by rats play a key role in research in this field, and the research results have significant implications for translational medicine. Although precise drug administration techniques such as TCI have been applied to dogs in some countries (such as Brazil), the existing equipment only supports a limited number of drugs (such as propofol and fentanyl) (15, 16). Therefore, developing rapid blood drug concentration measuring technologies applicable to more species and drugs is a crucial direction to fill the current research gap in veterinary medicine.

A novel portable mass spectrometer (CELL) has been developed to perform rapid quantitative analysis. Compared to HPLC-MS, CELL offers advantages in portability and speed, making it highly suitable for clinical settings that require rapid decision-making. Our previous work has preliminarily demonstrated its successful application in monitoring other drugs (17). Our team has previously investigated the application of portable mass spectrometry for monitoring rocuronium bromide concentrations in beagle plasma, establishing a foundational methodological framework. This study aims to evaluate the analytical performance of CELL and compare it with traditional HPLC-MS. This study evaluated the analytical performance of CELL, including specificity, linearity, and accuracy, for the quantitative determination of etomidate and dexmedetomidine. Moreover, it assesses the consistency between CELL and HPLC-MS in measuring plasma concentrations of these drugs in rats. The study further explores CELL's capability for real-time and simultaneous quantification of multiple drugs. The novel portable mass spectrometer demonstrates significant translational potential and holds promise for enabling more precise and individualized monitoring in both perioperative veterinary and human anesthesia.

## 2 Experimental methods

### 2.1 Analytical methodology validation

The validation of the analytical method was conducted in accordance with the guidelines established by the International Council for Harmonization of Technical Requirements for Pharmaceuticals for Human Use. Evaluation focused on key analytical performance parameters, including specificity, linear range, limit of detection (LOD), limit of quantification (LOQ), and accuracy (18).

### 2.2 Ethics approval

In this study, SPF-grade male Sprague-Dawley rats were used, weighing 280–300 g. All rats were purchased from Chengdu Dossy Experimental Animals Co., Ltd. (Chengdu, China). Prior to the experiment, all rats were acclimatized for 1 week under SPF conditions and constant temperature with food and water given *ad libitum*. All animal experiments were approved by the Animal Ethics Committee of West China Hospital, Sichuan University (Approval No. 20250723005) and strictly followed the NIH Guide for the Care and Use of Laboratory Animals and the ARRIVE guidelines.

### 2.3 Animal preparation

Tracheal intubation was performed, and all rats were connected to a ventilator (RWD, Shenzhen, China) via a re-breathing circuit with the following settings: an isoflurane concentration of 2.0%−2.5% mixed with an oxygen flow rate of 1.0 L/min, a respiratory rate of 50 breaths/min, and a tidal volume of 6 ml. Then, a 24G intravenous catheter (Surflo Flash, Terumo, Japan) was inserted into the left femoral artery for blood collection. Another 24G intravenous catheter (Surflo Flash, Terumo, Japan) was inserted into the tail vein for drug administration. Each rat received 2 ml of blood supplementation.

### 2.4 Drug administration

The 50% effective dose (ED_50_) of etomidate (Etomidate Injectable Emulsion, Nhwa Pharma, Xuzhou, China) and dexmedetomidine (Dexmedetomidine Hydrochloride Injection, Sichuan Meidakang Huakang Pharmaceutical, Deyang, China) were determined by the up-and-down method (19), with the criterion for judgment being the loss of the righting reflex (LORR) of the forelimbs in rats (20).

Eighteen rats were randomly assigned into three groups: the etomidate group (*n* = 6), the dexmedetomidine group (*n* = 6), and the mixed group receiving both etomidate and dexmedetomidine (*n* = 6). The sample size per group (*n* = 6) was determined through a careful integration of ethical and scientific considerations. First, this sample size adheres to the 3Rs principles, enabling the generation of pharmacologically meaningful results while minimizing animal usage. Furthermore, this study is exploratory. Rats in the etomidate group received a bolus injection of etomidate at a dose of 2 × ED_50_. Those in the dexmedetomidine group were administered a bolus injection of dexmedetomidine at 2 × ED_50_. In the mixed group, rats received a bolus injection containing both etomidate and dexmedetomidine at a dose of 2 × ED_50_ each.

### 2.5 Blood sample collection and analysis

#### 2.5.1 Sampling time points and sample pre-treatment

Blood samples of rats were collected at the following time points: before administration (0 min), and 1, 3, 5, 10, 15, 20, and 30 min after administration. At each time point, 0.3 ml of whole blood was collected. The whole blood was placed into an EP tube containing 10 μL of heparin (Heparin Sodium Injection, Hepa Tunn, Chengdu, China) and immediately stored on ice. After blood collection at all time points was completed, the whole blood was centrifuged using a centrifuge (Allegra 64R Centrifuge, Beckman Coulter, USA) with the following parameters: 3,500 rpm, 4 °C, and 10 min. After centrifugation, the upper plasma was transferred to a new EP tube for subsequent analysis.

#### 2.5.2 HPLC-MS detection

Etomidate detection: Took 50 μl of blank plasma, added 150 μl of IS (10 ng/ml ET impurity C in acetonitrile solution), vortexed, centrifuged at 20,000 rpm at 4 °C for 10 min, took the supernatant into an injection vial, and analyzed using HPLC-MS/MS (Agilent 1260 High Performance Liquid Chromatograph, Agilent G6460 Triple Quadrupole Mass Spectrometer, Agilent Mass-Hunter Workstation, USA). LC conditions: The chromatographic column was Agilent Extend C18 (3.0 × 100 mm, 3.5 μm). The mobile phase consisted of phase A (0.1% formic acid aqueous solution, Sigma-Aldrich, USA) and phase B (acetonitrile, Knowles, Chengdu, China) with a flow rate of 0.3 ml/min. The initial proportion of phase B was 16% and maintained for 2 min; then linearly increased to 20% within 2–2.5 min, further increased to 60% at 4 min, and raised to 90% at 6.5 min. The total running time was 6.5 min, followed by 2.5 min of column equilibration. The column temperature was maintained at 30 °C, and the injection volume was 1 μl. MS conditions: An electrospray ionization (ESI) source was used with positive ion detection mode and multiple reaction monitoring (MRM). The drying gas temperature was 350 °C with a flow rate of 5 L/min; the nebulizer pressure was set to 45 psi; the sheath gas temperature was 350 °C with a flow rate of 11 L/min; the capillary voltage was set to 3,500 V. The ion transition of etomidate was m/z 245 → 141, with a fragmentation voltage of 80 V and a collision energy of 4 V.

Dexmedetomidine detection: Took 50 μl of blank plasma, added 150 μl of IS (10 ng/ml dexmedetomidine-d4 in acetonitrile solution), vortexed, centrifuged at 20,000 rpm at 4 °C for 10 min, took the supernatant into an injection vial, and analyzed using HPLC-MS/MS (Agilent 1260 High Performance Liquid Chromatograph, Agilent G6460 Triple Quadrupole Mass Spectrometer, Agilent Mass-Hunter Workstation, USA). LC conditions: the chromatographic column was Agilent Extend C18 (3.0 × 100 mm, 3.5 μm). The mobile phase consisted of phase A (0.1% formic acid aqueous solution, Sigma-Aldrich, USA) and phase B (acetonitrile, Knowles, Chengdu, China) with a flow rate of 0.3 ml/min. The initial proportion of phase B was 25% and maintained for 3 min; then increased to 90% at 3.1 min. The total running time was 5 min, followed by 2.5 min of column equilibration. The column temperature was maintained at 30 °C, and the injection volume was 1 μl. MS conditions: an ESI source was used with positive ion detection mode and MRM. The drying gas temperature was 350 °C with a flow rate of 5 L/min; the nebulizer pressure was set to 45 psi; the sheath gas temperature was 350 °C with a flow rate of 11 L/min; the capillary voltage was set to 3,500 V. The ion transition of dexmedetomidine was m/z 201.2 → 95.1, with a fragmentation voltage of 108 V and a collision energy of 12 V.

Etomidate mixed with dexmedetomidine detection: Took 50 μl of blank plasma, added 150 μl of IS (acetonitrile mixed solution containing 25 ng/ml ET impurity C and 10 ng/ml dexmedetomidine-d4), vortexed, centrifuged at 20,000 rpm at 4 °C for 10 min, took the supernatant into an injection vial, and analyzed using HPLC-MS/MS (Agilent 1260 High Performance Liquid Chromatograph, Agilent G6460 Triple Quadrupole Mass Spectrometer, Agilent Mass-Hunter Workstation, USA). LC conditions: the chromatographic column was Agilent Extend C18 (3.0 × 100 mm, 3.5 μm). The mobile phase consisted of phase A (0.1% formic acid aqueous solution, Sigma-Aldrich, USA) and phase B (acetonitrile, Knowles, Chengdu, China) with a flow rate of 0.3 ml/min. The initial proportion of phase B was 16% and maintained for 2 min; then linearly increased to 20% within 2-2.5 min, further increased to 60% at 4 min, and raised to 90% at 7 min. The total running time was 7 min, followed by 2.5 min of column equilibration. The column temperature was maintained at 30 °C, and the injection volume was 1 μl. MS conditions: An ESI source was used with positive ion detection mode and MRM. The drying gas temperature was 350 °C with a flow rate of 5 L/min; the nebulizer pressure was set to 45 psi; the sheath gas temperature was 350 °C with a flow rate of 11 L/min; the capillary voltage was set to 3,500 V. The ion transition of etomidate was m/z 245 → 141, with a fragmentation voltage of 80 V and a collision energy of 4 V; the ion transition of dexmedetomidine was m/z 201.2 → 95.1, with a fragmentation voltage of 108 V and a collision energy of 12 V.

#### 2.5.3 CELL portable MS detection

The CELL portable mass spectrometer (C3001-sci, Suzhou Purspec Technology, Suzhou, China) is an analytical device based on tandem mass spectrometry, whose core components include an ion source, ion transmission system, linear ion trap, ion detection module, and control module. The physical parameters of the instrument are as follows: dimensions of 333 × 235 × 146 mm and weight not exceeding 8.5 kg; in terms of performance, its mass detection range covers 50–1,000 m/z, enabling switching between positive and negative ion modes, with the time for obtaining a single test result within 10 s.

The three groups of samples were processed using the same HPLC/MS method as described above. 100 μl of the treated supernatant was transferred to a direct capillary spray reagent kit (Suzhou Purspec Technology, Suzhou, China). Each sample was analyzed repeatedly 10 times in CELL. The total detection time for a single sample in the etomidate group and dexmedetomidine group was 2 min, while that in the etomidate mixed with dexmedetomidine group was 4 min. Etomidate: positive ion mode. The precursor ion of etomidate was m/z 245, the ionization energy was 1.5 V, and the fragment ion was m/z 141. Dexmedetomidine: positive ion mode. The precursor ion of dexmedetomidine was m/z 201.5, the ionization energy was 1.5 V, and the fragment ion was m/z 95.

### 2.6 Statistical analysis

Data analyses were performed using IBM SPSS Statistics 27 (IBM Corp., Armonk, NY, USA) and GraphPad Prism 10.4.1 (GraphPad Software Inc., San Diego, USA). The Shapiro–Wilk test was employed to evaluate data normality. Results are expressed as mean ± SD. The standard curves were constructed by fitting linear regression equations based on data from 3 independent analytical batches. Within the linear range, the theoretical concentrations of etomidate and dexmedetomidine served as the independent variable (*x*), and their peak heights were used as the dependent variable (*y*). The goodness of fit was evaluated using the coefficient of determination (*R*^2^), with *R*^2^ ≥ 0.99 indicating a good linear relationship. A 95% confidence interval (CI) was applied to test the statistical significance of the y-intercept of the regression equation. If 0 was contained within the 95% CI, the y-intercept was deemed statistically insignificant. The consistency between CELL and HPLC-MS was compared using intraclass correlation coefficient (ICC) analysis, and a result >85% was considered indicative of good consistency.

## 3 Results

### 3.1 Results of ED_50_

The ED_50_ of etomidate in rats measured by the up-and-down method was 0.9 mg/kg (95% CI: 0.85 to 0.99). The ED_50_ of dexmedetomidine was 21.1 μg/kg (95% CI: 19.6 to 22.7) (Supplementary Tables 1, 2).

### 3.2 Analytical methodology validation

#### 3.2.1 Specificity

**Figure 1:**
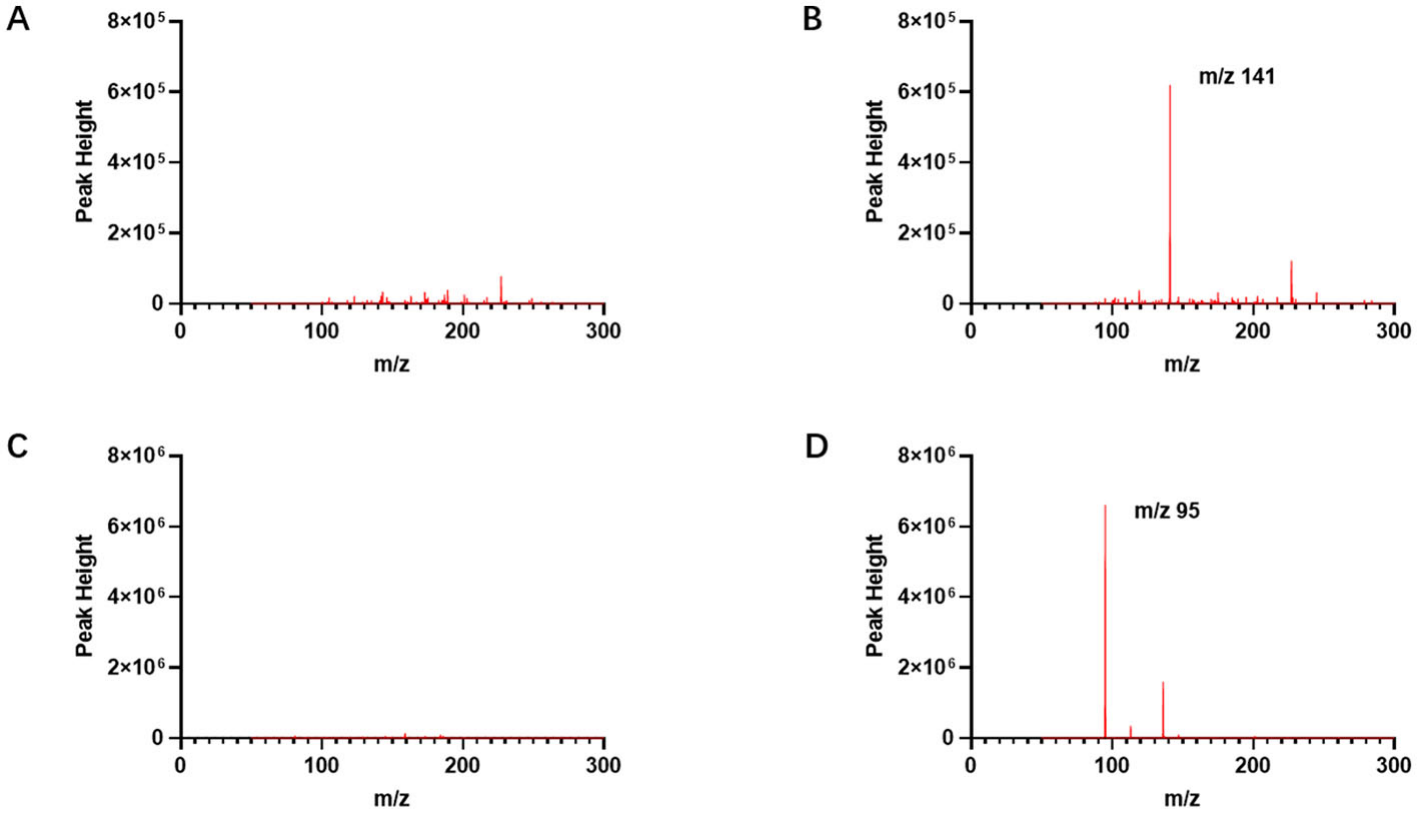
Full-scan mass spectrum of etomidate and dexmedetomidine measured by CELL portable mass spectrometer. **(A)** Presents the full-scan mass spectrum of a blank blood sample by the etomidate CELL method. **(B)** Presents the full-scan mass spectrum of a blank blood sample spiked with etomidate, with the fragment ion of etomidate being m/z 141. **(C)** Presents the full-scan mass spectrum of a blank blood sample by the dexmedetomidine CELL method. **(D)** Presents the full-scan mass spectrum of a blank blood sample spiked with dexmedetomidine, with the fragment ion of dexmedetomidine being m/z 95.

As shown in Figure 1, when comparing the full-scan mass spectra of blank blood samples obtained using the etomidate and dexmedetomidine CELL detection methods, respectively, it can be observed that the full-scan mass spectra of blank blood samples spiked with etomidate and dexmedetomidine exhibit significantly higher peak areas at their corresponding fragment ions (Figure 1). These results indicate that no significant interference was present at the detection ions for etomidate (m/z 141) and dexmedetomidine (m/z 95).

#### 3.2.2 Linear range

**Figure 2:**
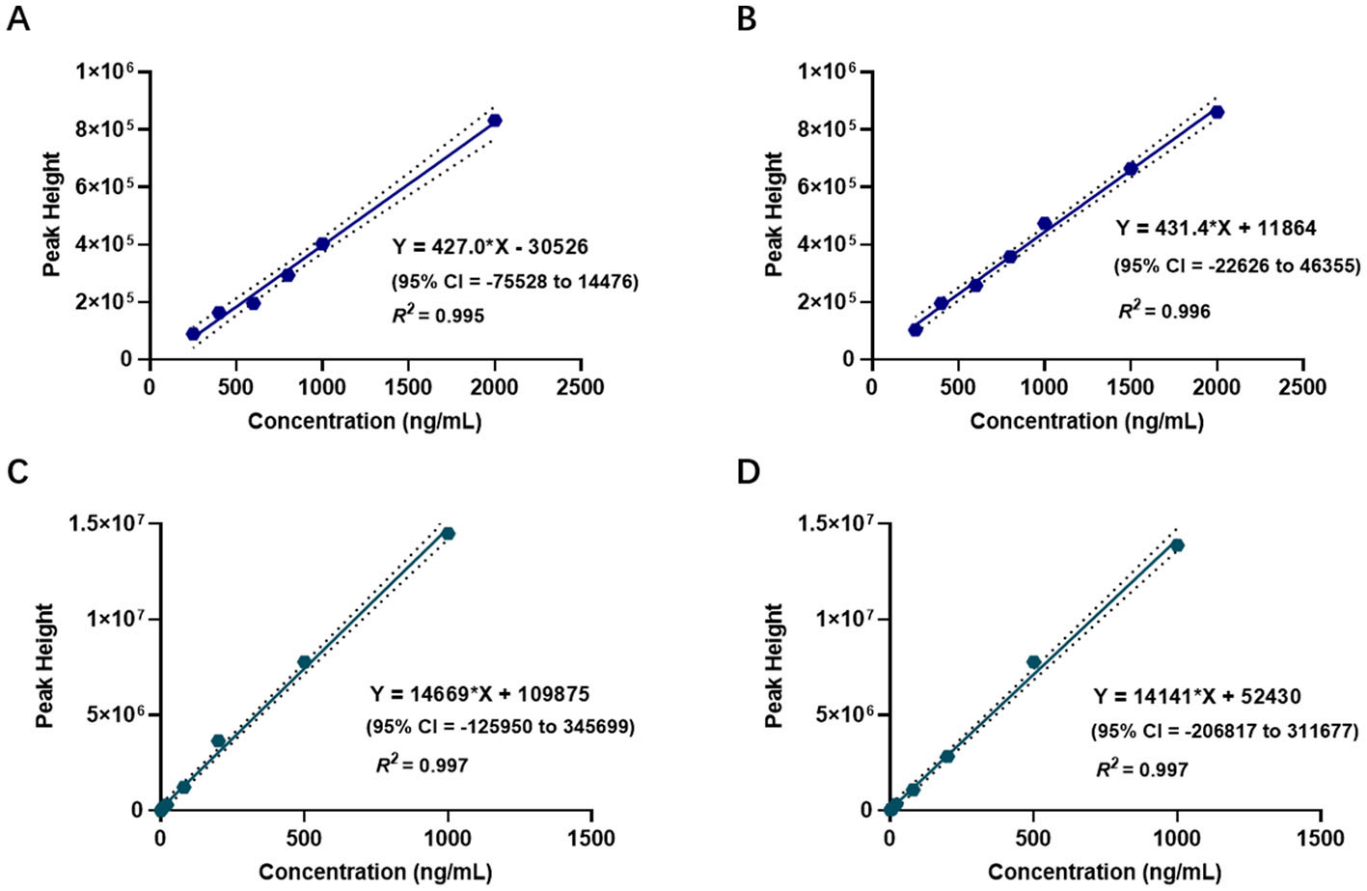
Linear range of etomidate and dexmedetomidine measured by CELL portable mass spectrometer. **(A)** Presents the linear range of the etomidate group. **(B)** Presents the linear range of the dexmedetomidine group. **(C)** Presents the linear range of etomidate in the mixed group. **(D)** Presents the linear range of dexmedetomidine in the mixed group.

The linear range of the etomidate group was determined using standard samples at concentrations of 250, 400, 600, 800, 1,000, and 2,000 ng/ml. As shown in Figure 2A, least squares regression was performed with the theoretical concentration as the independent variable (*x*) and the peak height at m/z 141 as the dependent variable (y). The results yielded a regression equation of *y* = 427.0*x* – 30,526 (95% CI = −75,528 to 14,476), with an *R*^2^ of 0.995, indicating a strong linear relationship within the concentration range of 210–2,000 ng/ml (Figure 2A).

The linear range of the dexmedetomidine group was determined using standard samples at concentrations of 1, 2, 4, 8, 20, 80, 200, 500, and 1,000 ng/ml. As shown in Figure 2B, least squares regression was conducted using the theoretical concentration as the independent variable (*x*) and the peak height at m/z 95 as the dependent variable (*y*). The results showed a regression equation of *y* = 14,669*x* + 109,875 (95% CI = −125,950 to 345,699), with an *R*^2^ of 0.997, demonstrating a strong linear relationship within the concentration range of 6–1,000 ng/ml (Figure 2B).

The linear range of the mixed group was evaluated using etomidate standard samples at concentrations of 250, 400, 600, 800, 1,000, 1,500, and 2,000 ng/ml, and dexmedetomidine standard samples at concentrations of 1, 2, 4, 8, 20, 80, 200, 500, and 1,000 ng/ml. As shown in Figures 2C, D, the regression equation for etomidate was *y* = 431.4*x* + 11,864 (95% CI = −22,626 to 46,355), with an *R*^2^ of 0.996, indicating a strong linear relationship within the concentration range of 100–2,000 ng/ml. For dexmedetomidine, the regression equation was y = 14141x + 52,430 (95% CI = −206,817 to 311,677), with an *R*^2^ of 0.997, indicating a strong linear relationship within the concentration range of 5–1,000 ng/ml (Figures 2C, D).

#### 3.2.3 LOD and LOQ

For the etomidate group, the average background noise at m/z 141 in blank samples was 5,796. The limit of detection (LOD), based on a signal-to-noise ratio of 3, was determined to be 120 ng/ml, while the limit of quantification (LOQ), corresponding to a signal-to-noise ratio of 10, was 210 ng/ml.

For the dexmedetomidine group, the average background noise at m/z 95 in blank samples was 11,375. The LOD, calculated using a signal-to-noise ratio of 3, was found to be 2 ng/ml, and the LOQ, based on a signal-to-noise ratio of 10, was 6 ng/ml.

In the mixed group, the average background noise of etomidate at m/z 141 was 5,138. The LOD, determined at a signal-to-noise ratio of 3, was 10 ng/ml, and the LOQ, at a signal-to-noise ratio of 10, was 100 ng/ml. For dexmedetomidine in the mixed group, the average background noise at m/z 95 was 11,822. The LOD was 2 ng/ml at a signal-to-noise ratio of 3, and the LOQ was 5 ng/ml at a signal-to-noise ratio of 10.

#### 3.2.4 Accuracy

Samples at three concentrations (low: 500 ng/ml, medium: 1,000 ng/ml, high: 1,500 ng/ml) were prepared to evaluate the accuracy of etomidate, with six parallel samples at each concentration used for analysis. The results indicated that the accuracy range for the etomidate group was 86.3%−98.4%, while for the etomidate in the mixed group it was 87.6%−104.0%. Additionally, samples at three concentrations (low: 25 ng/ml, medium: 200 ng/ml, high: 750 ng/ml) were prepared to assess the accuracy of dexmedetomidine. The results demonstrated that the accuracy range for the dexmedetomidine group was 88.5%−98.6%, and for the dexmedetomidine in the mixed group it was 96.3%−104.4%.

#### 3.2.5 Consistency comparison

**Figure 3:**
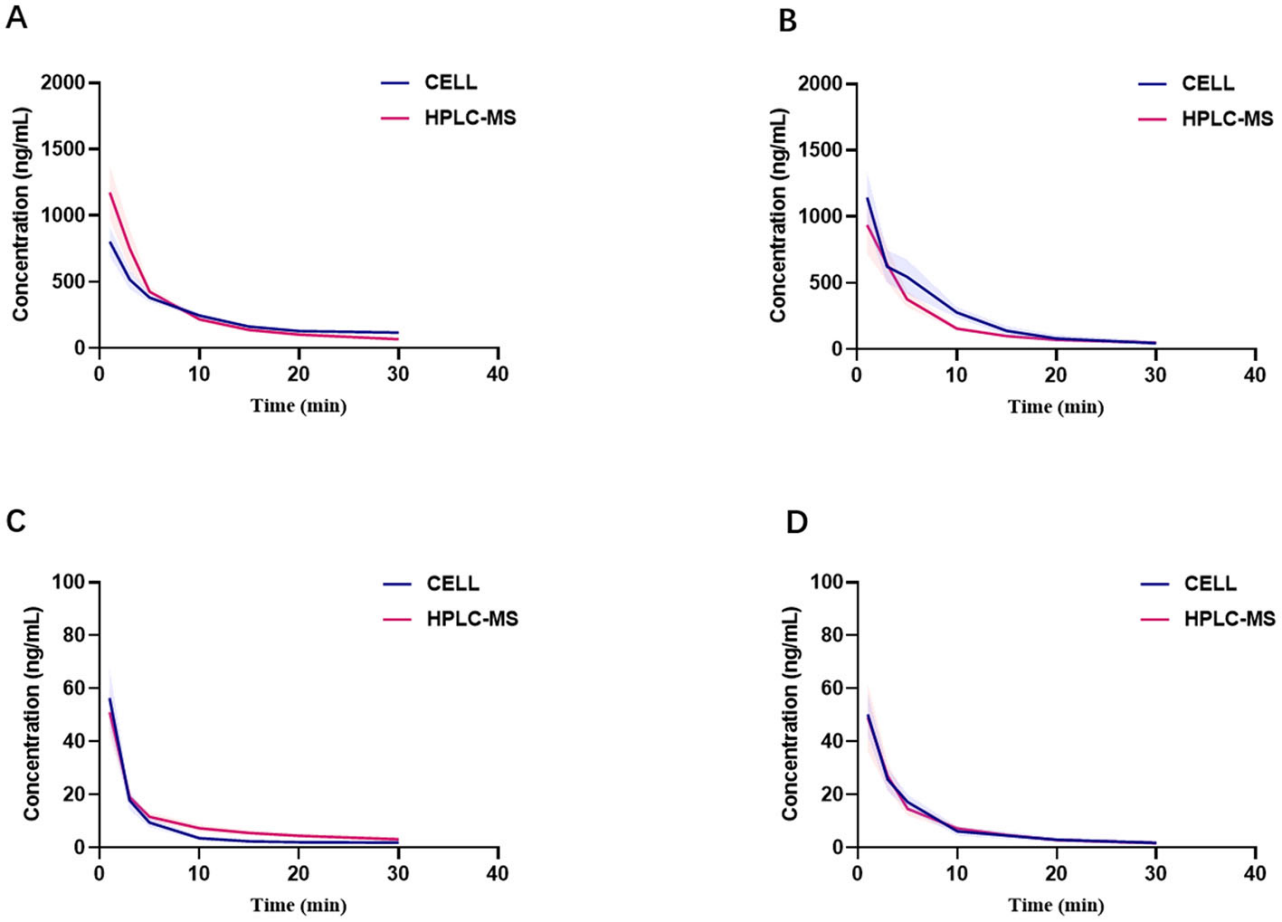
Comparison of blood concentrations measured by CELL portable mass spectrometer and HPLC-MS. **(A)** Presents blood concentration-time curves of etomidate group (*n* = 6). **(B)** Presents blood concentration-time curves of etomidate in the mixed group (*n* = 6). **(C)** Presents blood concentration-time curves of dexmedetomidine group (*n* = 6). **(D)** Presents blood concentration-time curves of dexmedetomidine in the mixed group (*n* = 6).

Figures 3A–D present the blood concentration-time curves of the three groups measured by HPLC-MS and CELL. The curves demonstrate that, following a single injection of etomidate and/or dexmedetomidine and until the drug effects subside, the variation trends of blood drug concentrations in all rats align with the fundamental principles of drug metabolism (Figures 3A–D).

**Figure 4:**
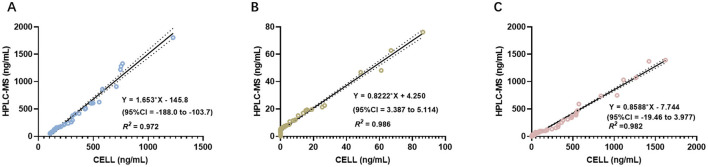
ICC analysis comparing the concentrations measured by CELL portable mass spectrometer and HPLC-MS. **(A)** Presents of the results of ICC analysis in the etomidate group. **(B)** Presents of the results of ICC analysis in the dexmedetomidine group. **(C)** Presents of the results of ICC analysis in the mixed group.

Figures 4A–C display the linear regression analyses of blood drug concentrations obtained using the two methods across the three groups. For the etomidate group, the linear regression equation was *Y* = 1.653^*^X – 145.8 (95% CI: −188.0 to −103.7), with an *R*^2^ of 0.972. For the dexmedetomidine group, the equation was *Y* = 0.8222^*^X + 4.250 (95% CI: 3.387 to 5.114), with an *R*^2^ of 0.986. For the mixed group, the equation was *Y* = 0.8588^*^X – 7.744 (95% CI: −19.46 to 3.977), with an *R*^2^ of 0.982. The results across all three groups exhibited strong consistency between the two measurement methods (Figures 4A–C).

Subsequently, ICC analysis was conducted to further assess the consistency between HPLC-MS and CELL. The Cronbach's α coefficient was 0.918 for the etomidate group, 0.985 for the dexmedetomidine group, and 0.990 for the mixed group. All Cronbach's α values exceeded 0.85, confirming substantial consistency between the two analytical methods (Table 1).

**Table 1:** Analytical methodology validation parameters for each group.

**Groups**	**Linear range**	**LOD**	**LOQ**	**Accuracy**	**Consistency comparison**	
					**Linear regression analyses**	**ICC analysis**
Etomidate	*y* = 427.0*x* −30526 (*R*^2^ = 0.995)	120 ng/ml at a signal-to-noise ratio of 3	210 ng/ml at a signal-to-noise ratio of 10	86.3%−98.4%	*R*^2^ = 0.972	Cronbach's α coefficient = 0.918
Dexmedetomidine	*y* = 14669*x* + 109875 (*R*^2^ = 0.997)	2 ng/ml at a signal-to-noise ratio of 3	6 ng/ml at a signal-to-noise ratio of 10	88.5%−98.6%	*R*^2^ = 0.986	Cronbach's α coefficient = 0.985
Etomidate in the mixed group	*y* = 431.4*x* + 11864 (*R*^2^ = 0.996)	10 ng/ml at a signal-to-noise ratio of 3	100 ng/ml at a signal-to-noise ratio of 10	87.6%−104.0%	*R*^2^ = 0.982	Cronbach's α coefficient = 0.990
Dexmedetomidine in the mixed group	*y* = 14141*x* + 52430 (*R*^2^ = 0.997)	2 ng/ml at a signal-to-noise ratio of 3	5 ng/ml at a signal-to-noise ratio of 10	96.3%−104.4%		

## 4 Discussion

This study aimed to evaluate the analytical performance of the CELL portable mass spectrometer and assess its consistency with the gold standard method, HPLC-MS, in measuring the blood concentrations of etomidate and dexmedetomidine. The operational principle of CELL is as follows: after ionization by the ion source, the generated ions are transported through the ion transmission system into the linear ion trap. Within the ion trap, ions are stored, selected, and fragmented before being detected in order of their mass-to-charge ratio. Our findings demonstrated that CELL exhibits excellent analytical performance and shows a high degree of consistency with HPLC-MS. Furthermore, this study not only confirmed the real-time detection capability of CELL but also validated its efficiency in simultaneously detecting a combination of etomidate and dexmedetomidine, highlighting its advantages in real-time analysis, ease of operation, and multi-drug detection capacity.

In this study, the up-and-down method was employed to determine the ED_50_ of etomidate and dexmedetomidine. A study by Zhu et al. (21) reported that the ED_50_ of etomidate administered via tail vein injection in rats was 0.74 mg/kg, whereas our result was 0.9 mg/kg. This discrepancy may be attributed to differences in the criteria used to assess LORR: Zhu et al. used the loss of righting reflex in all four limbs as the criterion, whereas this study used the loss of righting reflex in the forelimbs. For dexmedetomidine, Salonen et al. (22) reported an ED_50_ of 16.4 μg/kg, while our result was 20.1 μg/kg, which may be due to the use of a rotating cage to assess LORR in their study. Additionally, variations in laboratory environments may influence behavioral test outcomes, as previous studies have shown that rat behavior is closely related to environmental conditions (23, 24). Considering rat weight, species characteristics, and clinical drug administration purposes, this study adopted a dose of 2 × ED_50_ for experimental use (25, 26). Although this study selected a dose of 2 × ED_50_ based on these factors, the results highlight the necessity for future research focused on standardizing ED_50_ determination. Key areas for investigation include implementing standardized environmental controls, defining precise animal model specifications (e.g., strain, weight range), and validating behavioral testing protocols across laboratories to enhance reproducibility and translational validity.

The selection of etomidate and dexmedetomidine was based on their common use in sedation during anesthesia induction and maintenance. Their combined administration allows for dose reduction and minimizes the risk of hemodynamic instability (27, 28). In addition, dexmedetomidine is widely used in veterinary anesthesia, especially in small animal surgeries. It can help maintain the stability of respiratory and circulatory functions and provide appropriate sedation effects (29). In the study by Cui et al., the blood concentration of dexmedetomidine in rats was determined by HPLC-MS, with the reported C_max_ being 23.24 ng/ml. In contrast, the C_max_ values of dexmedetomidine measured by HPLC-MS and CELL in this study were 62.78 ng/ml and 67.03 ng/ml, respectively, which were significantly higher than the aforementioned results. This difference might be related to the setting of the blood sampling time points: the first blood sampling time in the Cui study was 2 min after administration, while in this study, the first blood sampling was advanced to 1 min after administration. Since the drug is directly injected into the systemic circulation via the tail vein, the early blood sampling through the femoral artery in this study enabled a more accurate capture of the initial peak of the drug concentration in the plasma, thereby obtaining a *C*_max_ value closer to the actual situation (30).

The linear dynamic range achieved by the CELL method was found to be appropriate for clinical applicability. Specifically, for etomidate, the linear range spanned from 100 to 2,000 ng/ml, while for dexmedetomidine, it extended from 5 to 1,000 ng/ml. In both cases, the *R*^2^ exceeded 0.99, indicating excellent linearity across the measured intervals. These ranges, which adequately encompass the established human therapeutic windows for effective anesthesia (31, 32), are particularly advantageous for veterinary applications. The high correlation coefficients, combined with broad linear dynamic range, demonstrate that the CELL provides a robust platform for quantitative analysis. This reliability is critical for intraoperative monitoring in small animals, where inter-individual variability and narrow safety margins of anesthetic agents pose significant challenges (33, 34). The method's capacity to accurately quantify drug concentrations across a wide range directly supports individualized dosing strategies, thereby enhancing surgical safety and advancing veterinary anesthesiology. Therefore, CELL has become a highly promising tool for rapid and accurate drug concentration monitoring. Its suitability for POC testing during the perioperative period holds significant translational potential in clinical trials and dose titration.

Currently, the monitoring of anesthetic drugs primarily relies on HPLC-MS (35). However, HPLC-MS presents several limitations, including bulky equipment, labor-intensive sample preparation, and complex operational procedures, which necessitate specialized personnel and restrict its use to laboratory settings. These constraints have limited the applicability of HPLC-MS in POC scenarios. POC diagnostics require mass spectrometers and analytical workflows that are user-friendly and suitable for on-site clinical use (36). In recent years, research on rapid detection of anesthetic drugs has mainly focused on illicit substances such as fentanyl. For instance, Fedick et al. integrated paper-based surface-enhanced Raman scattering with paper spray mass spectrometry on field-portable and commercial off-the-shelf devices to enable rapid and cost-effective identification of fentanyl and its analogs at suspicious scenes (37). In contrast, studies on real-time detection of general anesthetics and sedatives remain limited.

This study has several limitations that should be acknowledged. First, only the binary combination of two anesthetic agents was evaluated. Future investigations should incorporate more complex polypharmacy scenarios, such as the concurrent use of opioids and sedative-hypnotics, to better simulate real-world clinical anesthesia practices and more thoroughly assess potential pharmacodynamic and pharmacokinetic interactions. Second, all experiments were conducted using a rat model. Given the well-documented interspecies differences in drug metabolism and physiological responses, extrapolation of these findings to humans must be approached with caution. To enhance translational relevance, future studies should employ larger animal models—such as dogs or non-human primates—that more closely resemble human physiology and clinical anesthetic management. Additionally, the blood sampling timeline in this experiment was relatively short, which was appropriate given the primary aim of comparing the performance of the CELL method with HPLC-MS. Furthermore, as the distribution half-life of etomidate in rats is only ~2–3 min (38), the sampling time range in this study is sufficient to capture its early *in vivo* distribution process. However, pharmacokinetic phases beyond 30 min, particularly the elimination phase, were not captured in this study. To comprehensively reflect the complete pharmacokinetic characteristics including the elimination phase, subsequent studies should further extend the observation time and increase the sampling time points, thereby more systematically evaluating the metabolic dynamics of different anesthetic drugs. Moreover, validation was carried out at ED_50_ doses, which represent clinically effective concentrations but do not encompass the full therapeutic range, including subtherapeutic or supratherapeutic levels that may occur in clinical practice. Future studies should employ a systematic and multilevel experimental approach: designing experiments with multiple dose gradients, including subtherapeutic and supratherapeutic doses, and performing dose titration.

## 5 Conclusions

This study utilized the CELL portable mass spectrometer to rapidly and quantitatively analyze the concentrations of etomidate and dexmedetomidine in rats, with results highly consistent with those obtained by HPLC-MS. The CELL demonstrates outstanding performance in rapid drug quantification, enabling real-time analysis and simultaneous detection of two drugs. It is well-suited for the complex drug administration scenarios in clinical anesthesia, and holds significant value in precise TCI, maintaining stable vital signs during surgery, and optimizing drug dosages.

## Data Availability

The raw data supporting the conclusions of this article will be made available by the authors, without undue reservation.
