# Supplementary material for: Rapid quantification and validation of etomidate and dexmedetomidine blood concentrations in rats using a novel portable mass spectrometer
Source: Front Vet Sci. 2025 Oct 29;12:1696093. doi: 10.3389/fvets.2025.1696093 (PMC12605077; doi:10.3389/fvets.2025.1696093)
Supplement: Supplementary file 1 [file Table_1.docx]

Supplementary Material

Rapid Quantification and Validation of Etomidate and Dexmedetomidine Blood Concentrations in Rats Using a Novel Portable Mass Spectrometer

Sicheng Liu^1,2,†^, Xiaoxiao Li^1,2,†^, Deying Gong^2^, Yanhua Huang^2^, Wensheng Zhang^1,2,*^

^1^Department of Anesthesiology, West China Hospital, Sichuan University, Chengdu, China,

^2^Laboratory of Anesthesia and Critical Care Medicine, National-Local Joint Engineering Research

Centre of Translational Medicine of Anesthesiology, West China Hospital, Sichuan University,

Chengdu, China

*** Correspondence:**Wensheng Zhang

[zhang_ws@scu.edu.cn](mailto:zhang_ws@scu.edu.cn)

†These authors have contributed equally to this work

# Supplementary Figures and Tables

**Table1. Experimental results of ED_50_ for etomidate**

| **Dose-D***  **(mg/kg)** | **Logarithmic Dose-X***  **(log D)** | **Experimental Results (+/-)*** | | | | | | | | | | | | | | | | | | | | | **t value** | **C***  **(t×X)** | | **M***  **(X^2^t)** | |
| --- | --- | --- | --- | --- | --- | --- | --- | --- | --- | --- | --- | --- | --- | --- | --- | --- | --- | --- | --- | --- | --- | --- | --- | --- | --- | --- | --- |
|  |  | **1** | **2** | **3** | **4** | **5** | **6** | **7** | **8** | **9** | **10** | **11** | | **12** | **13** | **14** | **15** | **16** | **17** | **18** | **19** | **20** |  |  |  |  |  |
| 1.60 | 0.20 | **+** |  |  |  |  |  |  |  |  |  |  | |  |  |  |  |  |  |  | **+** |  | 2 | 0.41 | | | 0.08 |
| 1.28 | 0.11 |  | **+** |  |  |  |  |  |  |  |  |  | |  |  |  |  |  |  | **-** |  | **-** | 3 | 0.32 | | | 0.03 |
| 1.02 | 0.09 |  |  | **+** |  |  |  |  |  |  |  |  | |  | **+** |  | **+** |  | **-** |  |  |  | 4 | 0.03 | | | 0.00 |
| 0.82 | -0.09 |  |  |  | **+** |  | **+** |  |  |  | **+** |  | | **-** |  | **-** |  | **-** |  |  |  |  | 6 | -0.52 | | | 0.04 |
| 0.66 | -0.18 |  |  |  |  | **-** |  | **+** |  | **-** |  | **-** | |  |  |  |  |  |  |  |  |  | 4 | -0.72 | | | 0.13 |
| 0.52 | -0.28 |  |  |  |  |  |  |  | **-** |  |  |  | |  |  |  |  |  |  |  |  |  | 1 | -0.28 | | | 0.08 |
| **Total** | | | | | | | | | | | | | | | | | | | | | | | 20 | | -0.76 | | 0.37 |
| ED_50_= 0.9 mg/kg | | | | | | | | | | | | | 95%CI: 0.85 to 0.99 | | | | | | | | | | | | | | |

*: “+” indicates that the rat exhibits LORR, and “-” indicates that the rat does not exhibit LORR. “D” represents the dose of the drug; “X” represents the logarithm of “D”; “C” represents the product of t value and “X”; “M” represents the square of the product of t value and “X”. (n=20)

**Table2. Experimental results of ED_50_ for dexmedetomidine**

| **Dose-D***  **(μg/kg)** | **Logarithmic Dose-X***  **(log D)** | **Experimental Results (+/-)*** | | | | | | | | | | | | | | | **t value** | **C***  **(t×X)** | **M***  **(X^2^t)** |
| --- | --- | --- | --- | --- | --- | --- | --- | --- | --- | --- | --- | --- | --- | --- | --- | --- | --- | --- | --- |
|  |  | **1** | **2** | **3** | **4** | **5** | **6** | **7** | **8** | **9** | **10** | **11** | **12** | **13** | **14** | **15** |  |  |  |
| 13.3 | 1.12 |  |  |  |  | **-** |  |  |  |  |  |  |  | **-** |  | **-** | 3 | 3.37 | 3.80 |
| 20.0 | 1.39 |  | **+** |  | **+** |  | **-** |  | **-** |  | **-** |  | **+** |  | **+** |  | 7 | 9.11 | 11.8 |
| 30.0 | 1.48 | **+** |  | **+** |  |  |  | **+** |  | **+** |  | **+** |  |  |  |  | 5 | 7.39 | 10.9 |
| **Total** | | | | | | | | | | | | | | | | | 15 | 19.9 | 26.6 |
| ED_50_= 21.1μg/kg | | | | | | | | | | | 95%CI: 19.6 to 22.7 | | | | | | | | |

*: “+” indicates that the rat exhibits LORR, and “-” indicates that the rat does not exhibit LORR. “D” represents the dose of the drug; “X” represents the logarithm of “D”; “C” represents the product of t value and “X”; “M” represents the square of the product of t value and “X”. (n=15)
